# Case Report of a Tongue-Type Calcaneal Fracture

**DOI:** 10.21980/J8NH11

**Published:** 2023-01-31

**Authors:** Kylie T Callan, Michael Head, Gregg Pickett, Ronald Rivera

**Affiliations:** * University of California, Irvine, Department of Emergency Medicine, Orange, CA; ^ City of Surprise Fire-Medical Department, Sunrise, AZ

## Abstract

Calcaneus fractures make up only 2% of all fractures, and tongue-type calcaneus fractures represent 25–40% of all calcaneus fractures. While rare, tongue-type calcaneus fractures can put the superficial soft tissue at risk for necrosis and other complications, creating a surgical emergency. This case report describes the care of a patient who presented to a remote island critical-access emergency room after a fall from height. He described severe, sharp pain in the heel and was found to have tenting and blanching of the overlying skin near the injury. These findings suggested the soft tissues were at high risk for necrosis if not treated immediately. An X-ray confirmed a tongue-type calcaneus fracture. The patient was splinted in plantar flexion and transported by helicopter to a mainland hospital capable of performing the appropriate surgical reduction. The patient underwent open reduction internal fixation of the calcaneus, relieving pressure on the skin. He tolerated the procedure well, and there were no complications. He was discharged the day after surgery with outpatient follow-up. His case was prolonged and required multiple procedures to ultimately achieve appropriate healing. This case illustrates the importance of recognizing fractures presenting with skin involvement since surgical emergencies require prompt intervention to reduce the risk of serious complications such as open fracture from skin breakdown, poor healing, and a slow return to normal activities. It also emphasizes the importance of advocating for expedient patient care to increase the odds of a good outcome and ensure patients are given high-quality care.

**Topics:**

Calcaneus fracture, tongue-type calcaneus fracture, fall from height, axial loading, fracture complications, case report.

## Brief introduction

[Fig f4-jetem-8-1-v28][Fig f5-jetem-8-1-v28][Fig f6-jetem-8-1-v28][Fig f7-jetem-8-1-v28][Fig f8-jetem-8-1-v28][Fig f9-jetem-8-1-v28][Fig f10-jetem-8-1-v28][Fig f11-jetem-8-1-v28]Tongue-type calcaneus fractures are an uncommon fracture to see in the general population but represent a surgical emergency and must be rapidly and correctly identified to avoid complications. The bone fragments in this type of fracture tend to put pressure on the overlying skin and can cause rapid skin breakdown, leading to necrosis and possible open fracture. Tongue-type calcaneal fractures are longitudinal fractures that affect at least part of the articular surface and run through to the posterior aspect of the bone.^1^ Tongue-type fractures represent roughly 25–40% of all calcaneus fractures.^2^,^3^ Calcaneus fractures make up 60% of tarsal fractures and about 2% of all fractures.^1^–^4^ Calcaneal fractures have an annual incidence of around 11.5 per 100,000 people and are more common in males.^5^ They are most often due to axial loading forces or falls from a height, with a majority of calcaneal fractures occurring from falls greater than six feet.^4^,^5^ This type of fracture can have complications including delayed wound healing, infection, increased length of hospital stay, soft tissue necrosis, neurovascular compromise, and osteomyelitis, and must be treated with urgency.^1^,^3^,^6^ Rapid recognition of concerning factors and characteristics of tongue-type fractures is vital to ensuring good outcomes for patients. Written consent was obtained from the patient for publication of the photographs.

## Presenting concerns and clinical findings

The patient was a 49-year-old male visiting Catalina Island when he suffered a fall from the back row of a concrete amphitheater. He described a fall of approximately ten feet, where he landed on the lateral aspect of his right heel on concrete ground. He then fell sideways and rolled through the rest of the fall. The patient described constant, severe, sharp pain in the right heel after the fall, which made him unable to ambulate at the scene without assistance. He could not put weight on the affected foot due to pain. He did not strike his head, nor did not lose consciousness, and he had no back pain. His review of systems was grossly unremarkable, except for pain in the posterior ankle and nausea when the pain was worst. His vital signs were unremarkable throughout his presentation. His medical, family, and social history were noncontributory.

## Significant findings

Examination of the right ankle demonstrated a large deformity of the superior talus with bruising and blanching of the overlying skin in the area of the Achilles tendon (see images 2,3). The remaining bones of the foot were not tender to palpation and the foot was neurovascularly intact throughout with only mild numbness in the area of the tented skin. Completing the trauma exam, the patient had no signs of head injury and no midline spinal tenderness to palpation. Inspection of the remaining long bones and joints showed no other injuries. There were mild skin scrapes on the right flank from the fall. X-rays of the right foot and ankle showed a longitudinal fracture of the calcaneal tuberosity from the articular surface to the posterior surface (see red outline) with extension into the subtalar joint (blue lines) and roughly 1.8 cm displacement between the fracture segments (yellow double arrow). These findings represented a tongue-type calcaneal bone fracture.

## Patient course

Based on history, physical exam, and imaging, the diagnosis of tongue-type calcaneus fracture was made. A tongue-type calcaneal fracture is a longitudinal fracture that involves a portion of the articular surface and exits the calcaneal tuberosity posteriorly. The skin tenting, blanching, decreased sensation, and bruising suggested pressure from the fracture fragment on the skin was limiting perfusion and putting the tissue at risk of necrosis. This constellation of findings indicated a need for emergency surgery to preserve the tissue and limit complications. Since the patient was on an island at a critical-access emergency department (ED), resources were limited, with no orthopedic surgeons available to perform the surgery. To reduce pressure on the skin, the patient’s lower leg was wrapped in bulky padding with anterior and posterior splints placed to hold the foot in plantarflexion. The patient was then endorsed to an orthopedic surgeon and airlifted by helicopter to a mainland hospital where he could be taken to the operating room immediately. On arrival, he was taken for emergent open reduction internal fixation (ORIF) of the right calcaneus to relieve the stress on the skin and align the tongue fragments using three orthopedic screws. The patient tolerated the procedure well with no complications. He was discharged one day after surgery, non-weightbearing on the right lower extremity. Unfortunately, the patient’s post operative course was complicated by a fall in the shower which broke his original hardware, requiring two additional surgeries to repair. Fortunately, the patient recovered well after the third surgery, approximately 3 months after his original injury.

## Discussion

Tongue-type calcaneal fractures are rare and often occur in falls from height or during high-force axial loading.^4^,^5^ Patients typically present with acute, sharp, localized heel pain with deformity and blanching of the overlying skin. They may also have localized sensory deficits. There is usually gross deformity of the heel, but they can present more subtly. X-ray imaging must be obtained to make the diagnosis, but orthopedic surgery should be consulted early if local neurovascular compromise is suspected before imaging is available. This type of fracture warrants immediate orthopedic evaluation and intervention to prevent limb-threatening complications; therefore, it is imperative for the initial physician to recognize the above critical signs quickly and advocate for early surgical intervention.

**Image 1:**
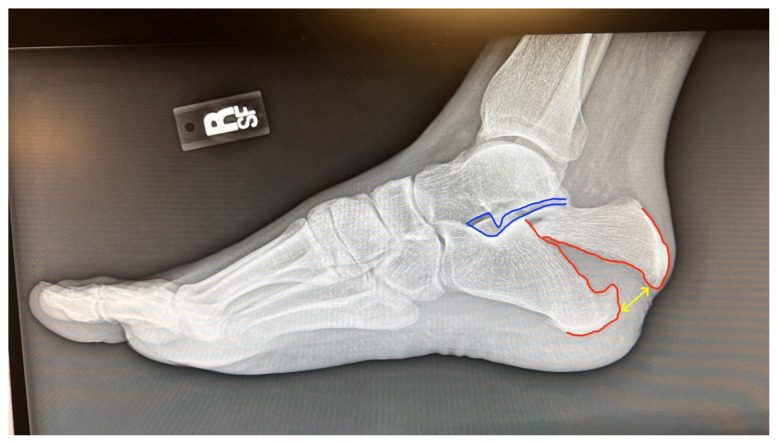
Initial X-Ray

**Image 2:**
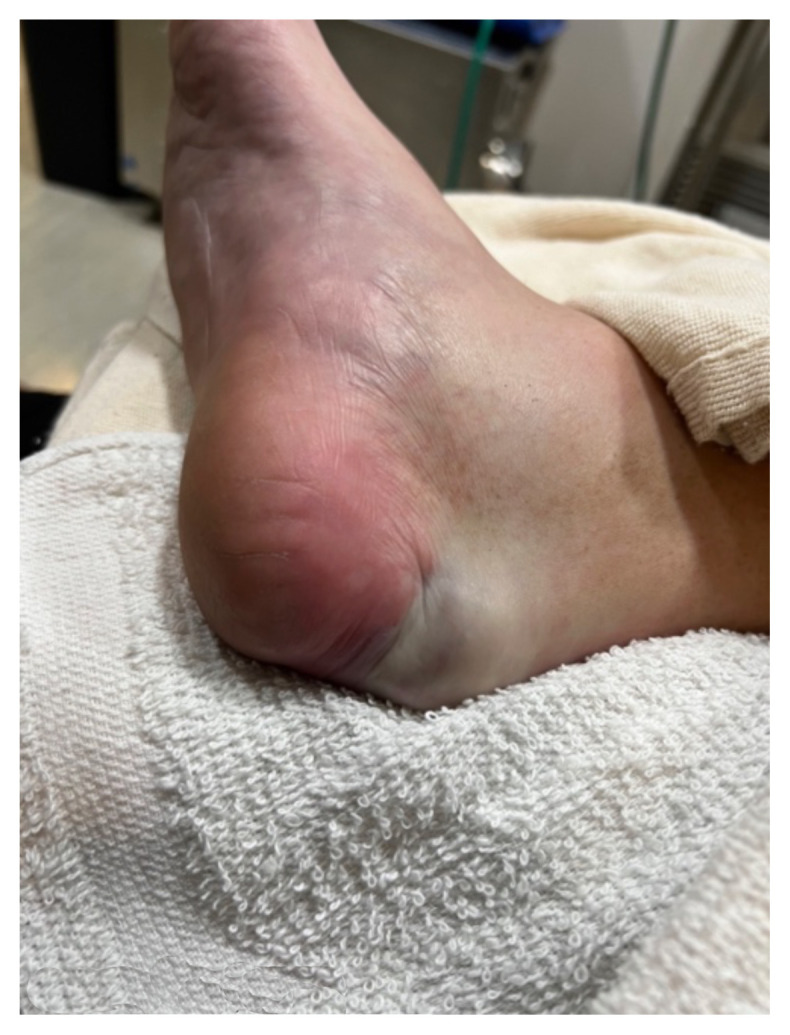
Initial Exam 1

**Image 3:**
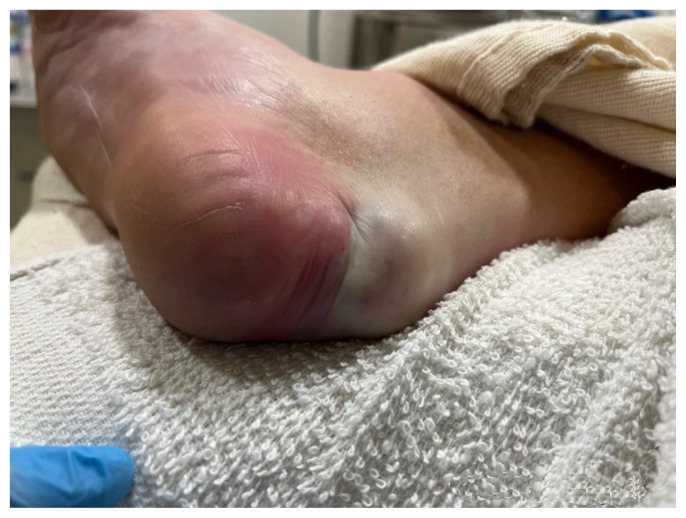
Initial Exam 2

Many of these findings were present in our patient, including the characterization of the pain, the blanching of the skin, and deformity of the heel. This diagnosis was further supported by the clear fracture findings as shown on X-ray (image 1). Data on the sensitivity and specificity of plain radiographs for diagnosis of calcaneal fractures is limited.^7^ Plain radiographs show enough to make a diagnosis, especially if there is clear displacement of the fracture fragments. The lateral view is considered to be more helpful than the axial or anteroposterior views for identifying calcaneal fractures in general.^7^,^8^ To confirm a non-obvious calcaneal fracture, one can calculate Bohler’s angle and the angle of Gissane using the lateral view, but the physician can usually rely on the radiologist to make this determination.^7^ If there is continued suspicion or the x-ray images are not clear, advanced imaging such as non-contrast computed tomography (CT) or magnetic resonance imaging (MRI) can be used to make the diagnosis if available.^4^,^7^

In this type of fracture, the risk of skin compromise increases with delay to presentation to the ED, as well as with increased fracture displacement.^9^ In this case, the ED physician needed to stabilize the fracture in an attempt to remove pressure from the skin to slow the damage. Left untreated, the skin would eventually become necrotic, opening a wound to an intra-articular fracture. This would put the patient at risk for bone and joint infections, poor healing of the fracture, a possible need for skin grafting to close the wound, and significantly higher risks for morbidity and mortality. Placing the foot in plantarflexion reduces tension on the Achilles tendon attached to the superior fracture fragment and allows a slight reduction of the bony fragment away from the skin. It is important to recognize that this is only a temporizing maneuver because it does not reduce the fracture enough to completely prevent skin necrosis. It is merely to protect the skin and an attempt to slow the necrosis until surgical treatment can be rendered. For operative treatment, one review found that there was no definite advantage of either closed or open reduction, although the authors recommended attempting a closed reduction if possible.^10^ In this case, the patient underwent an open procedure. The patient tolerated the procedure well and had a good outcome, despite his complicated course. Recovery time based on limited research indicates that fracture healing for this type of injury takes 8 weeks on average with a return to usual activity in an average of 4.3 months.^11^

## Supplementary Information













































## Figures and Tables

**Image 4 f4-jetem-8-1-v28:**
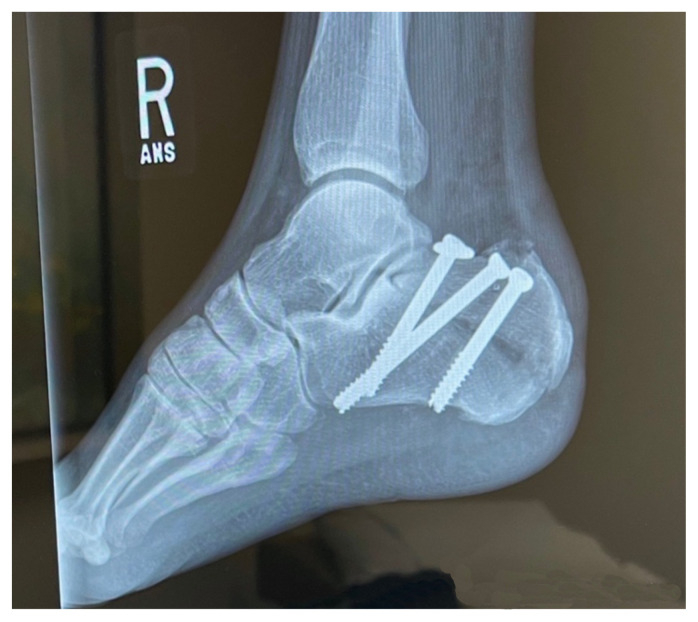
X-Ray After Initial Procedure

**Image 5 f5-jetem-8-1-v28:**
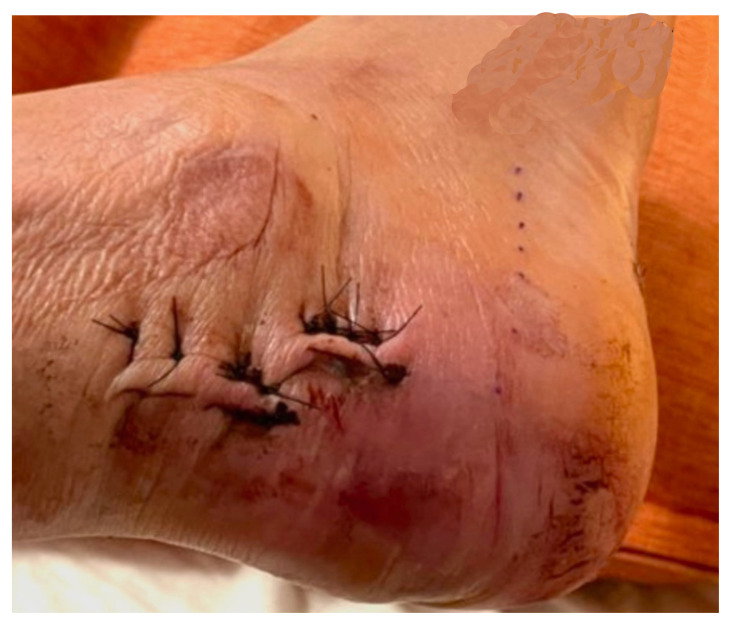
Posterior Ankle 9 Days After Initial Procedure

**Image 6 f6-jetem-8-1-v28:**
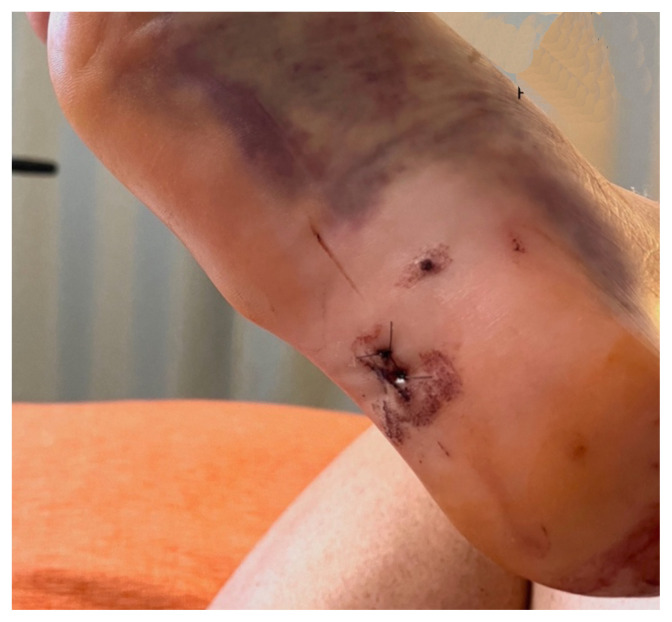
Plantar Aspect of Foot 9 Days after Initial Procedure

**Image 7 f7-jetem-8-1-v28:**
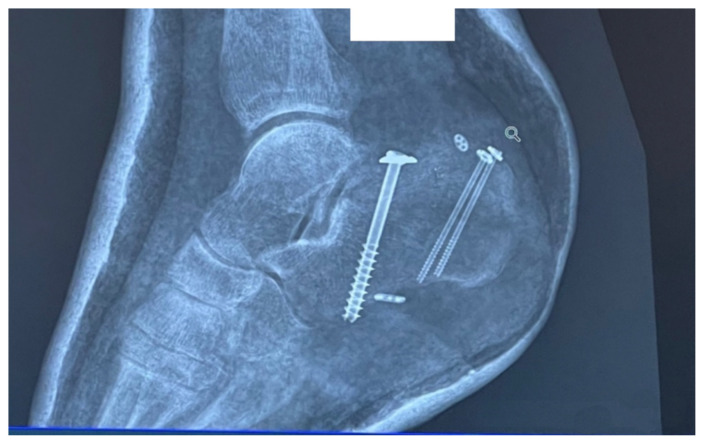
X-Ray After 3rd Surgery

**Image 8 f8-jetem-8-1-v28:**
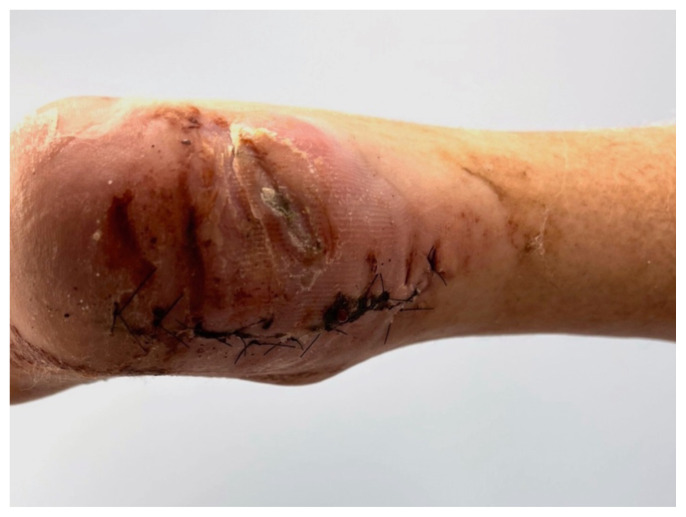
14 Days After 3rd Surgery

**Image 9 f9-jetem-8-1-v28:**
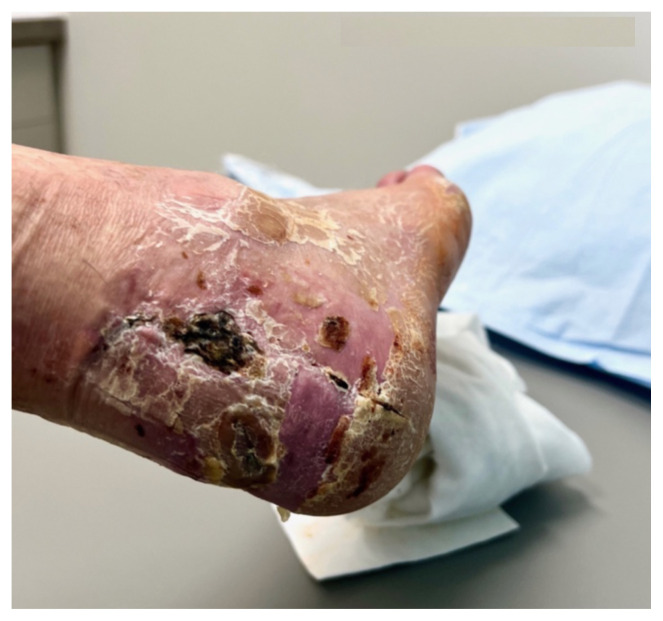
1 month After 3rd Surgery

**Image 10 f10-jetem-8-1-v28:**
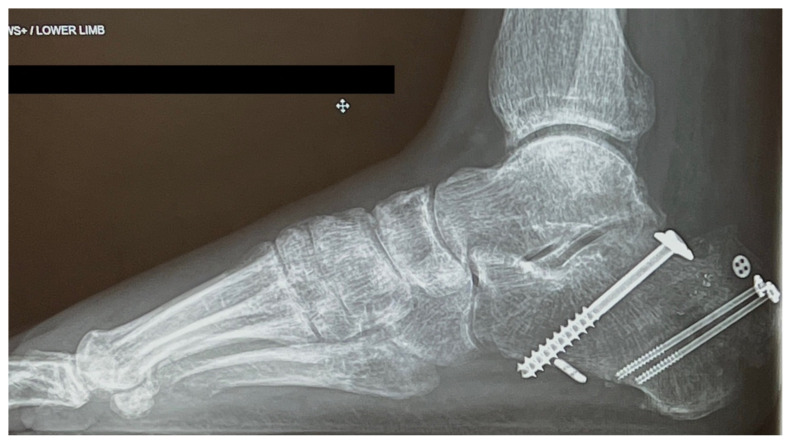
X-Ray 2 months After 3rd Surgery

**Image 11 f11-jetem-8-1-v28:**
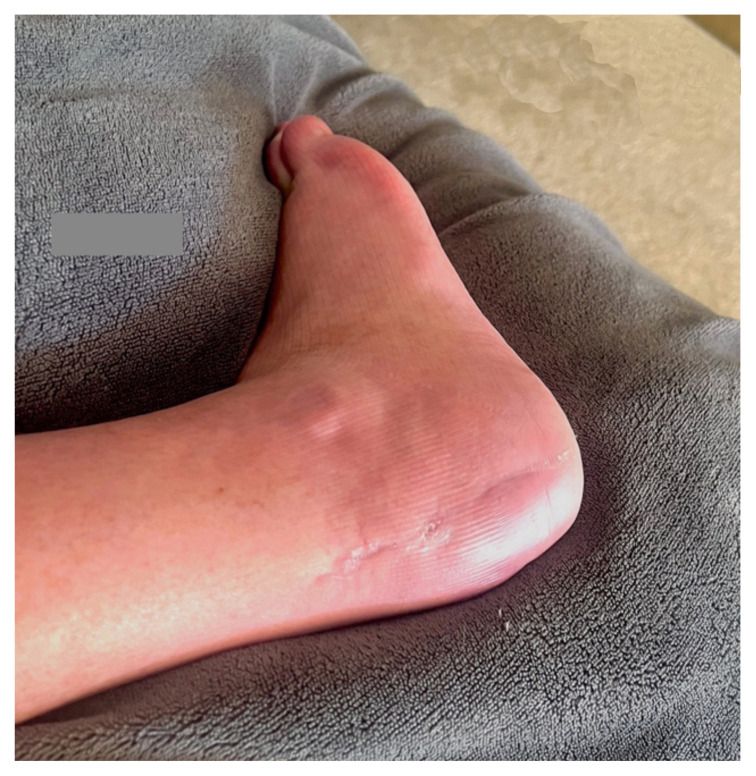
2 months After 3rd Surgery
