# Supplementary figures and images for: Case Report of a Tongue-Type Calcaneal Fracture
Source: J Educ Teach Emerg Med. 2023 Jan 31;8(1):V28–34. doi: 10.21980/J8NH11 (PMC10332765; doi:10.21980/J8NH11)

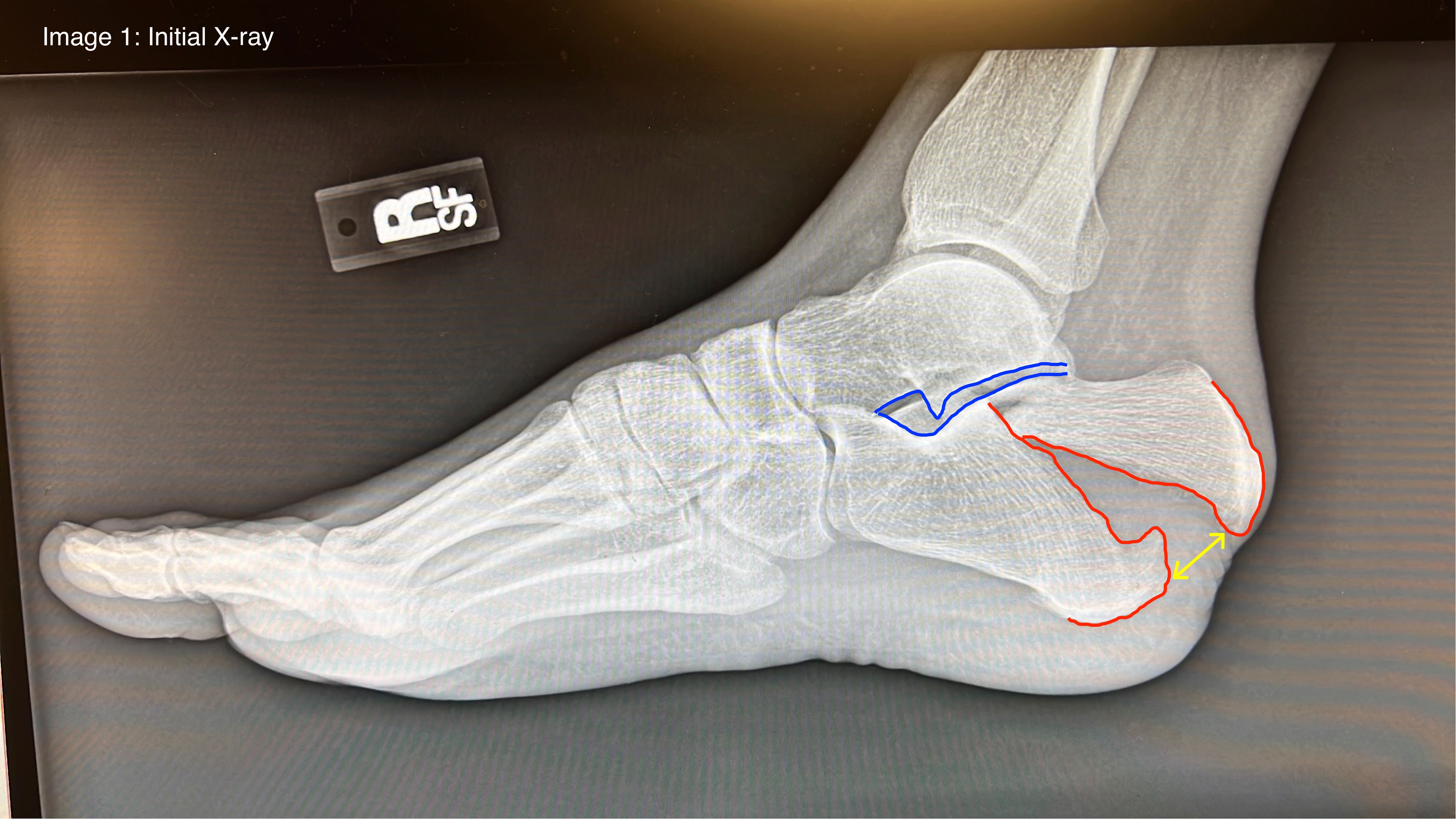

Supplement: Supplementary file 1 [file jetem-8-1-v28-supp1.jpg]

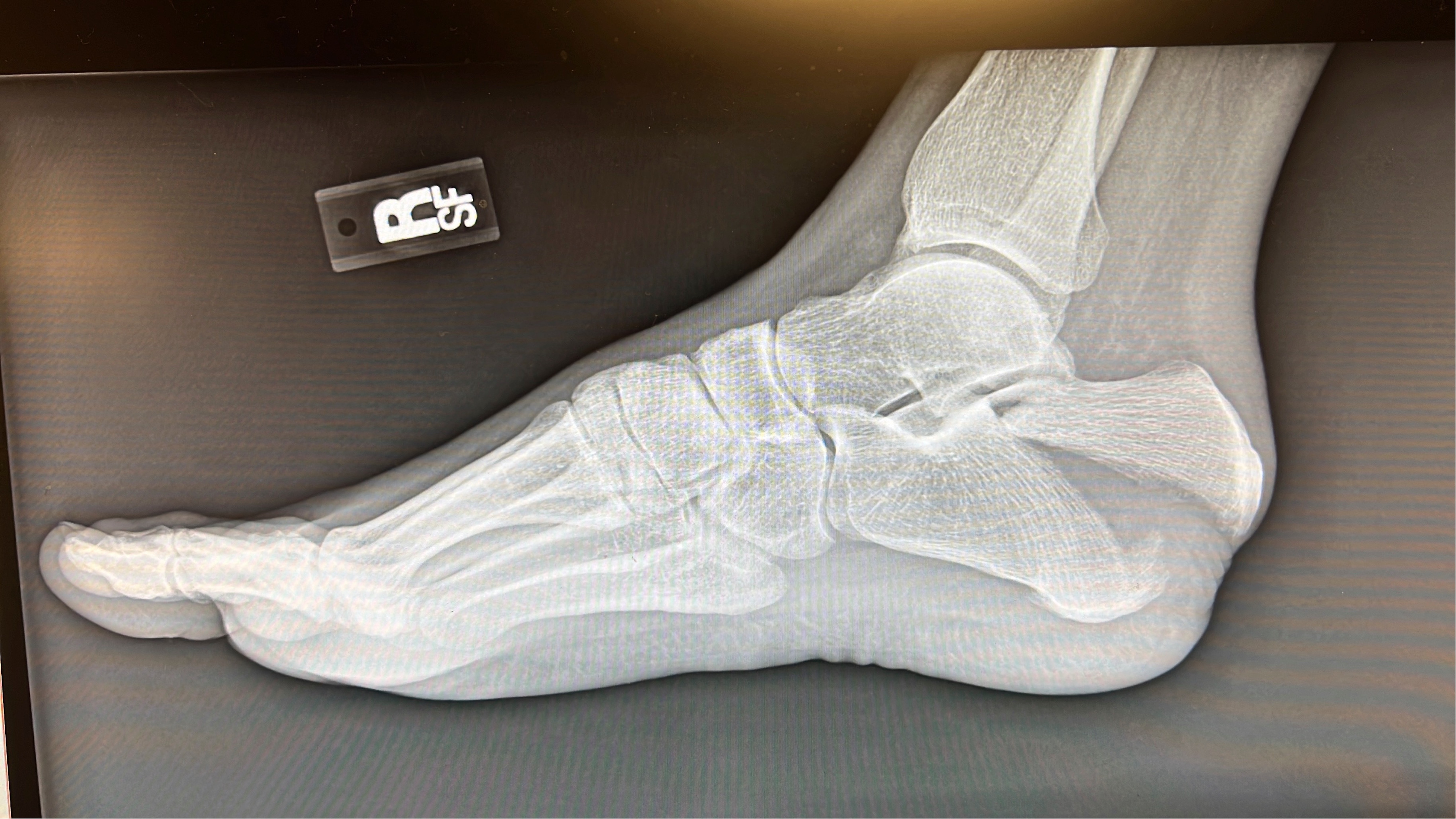

Supplement: Supplementary file 2 [file jetem-8-1-v28-supp2.jpg]

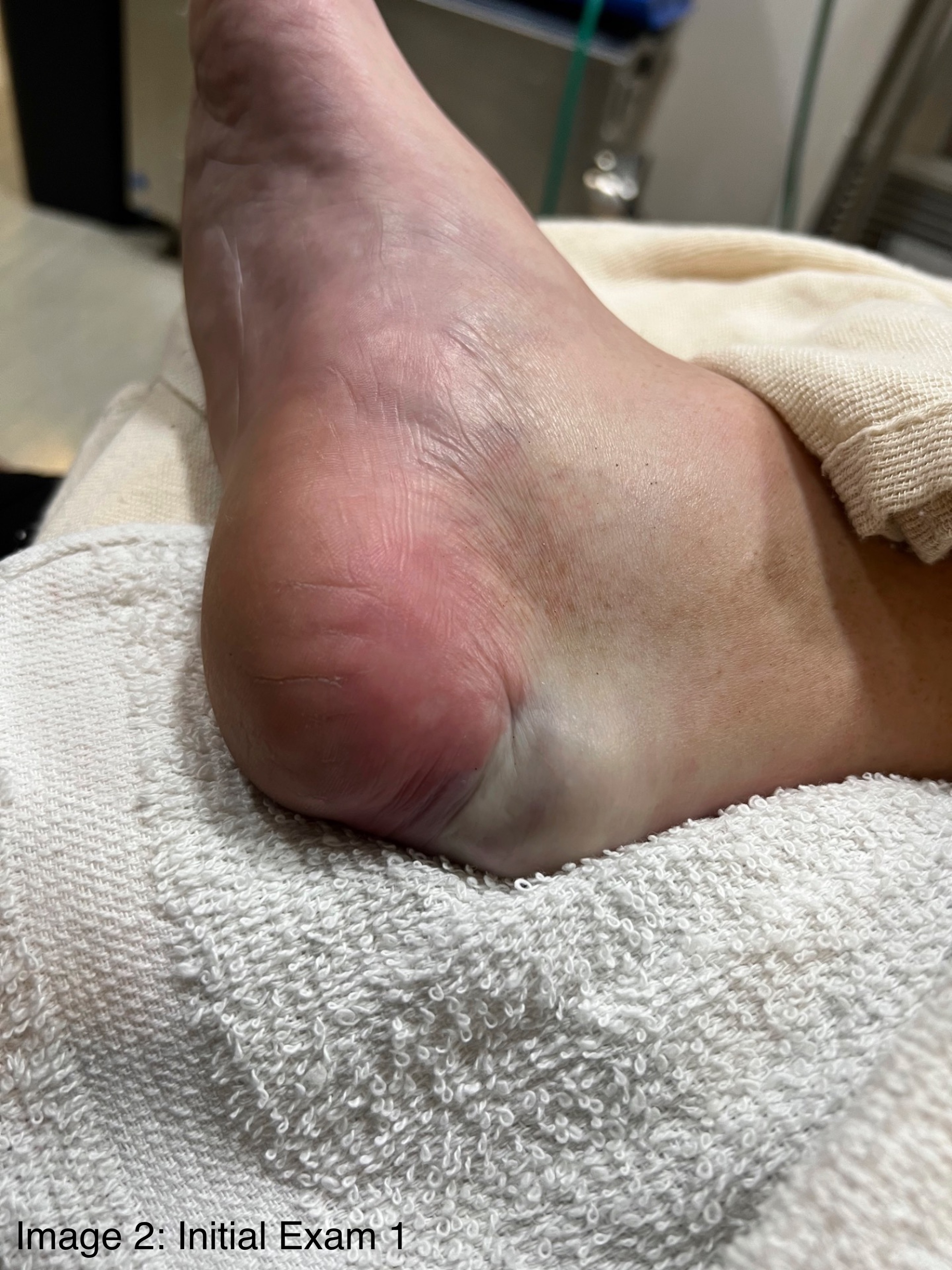

Supplement: Supplementary file 3 [file jetem-8-1-v28-supp3.jpg]

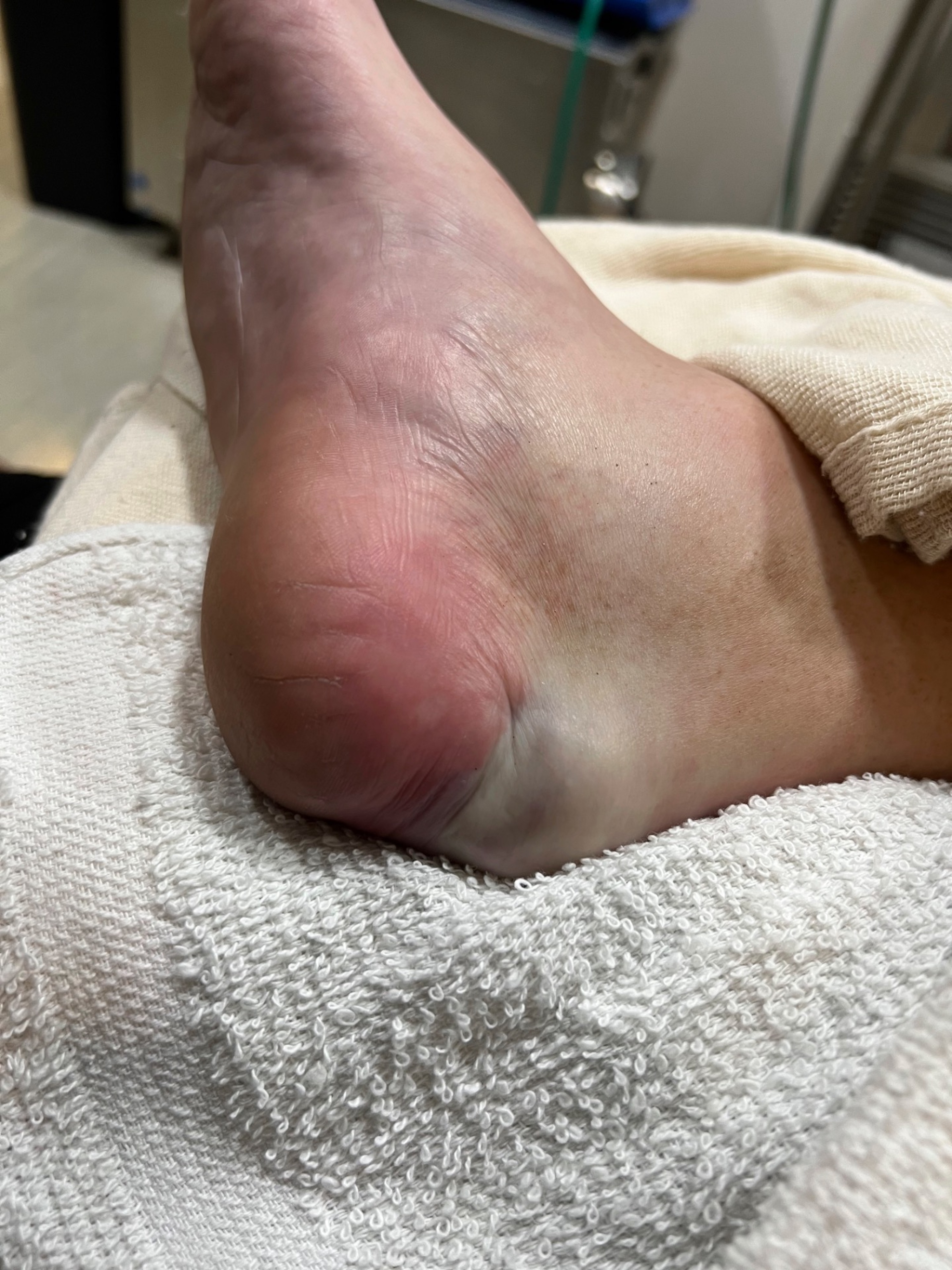

Supplement: Supplementary file 4 [file jetem-8-1-v28-supp4.jpg]

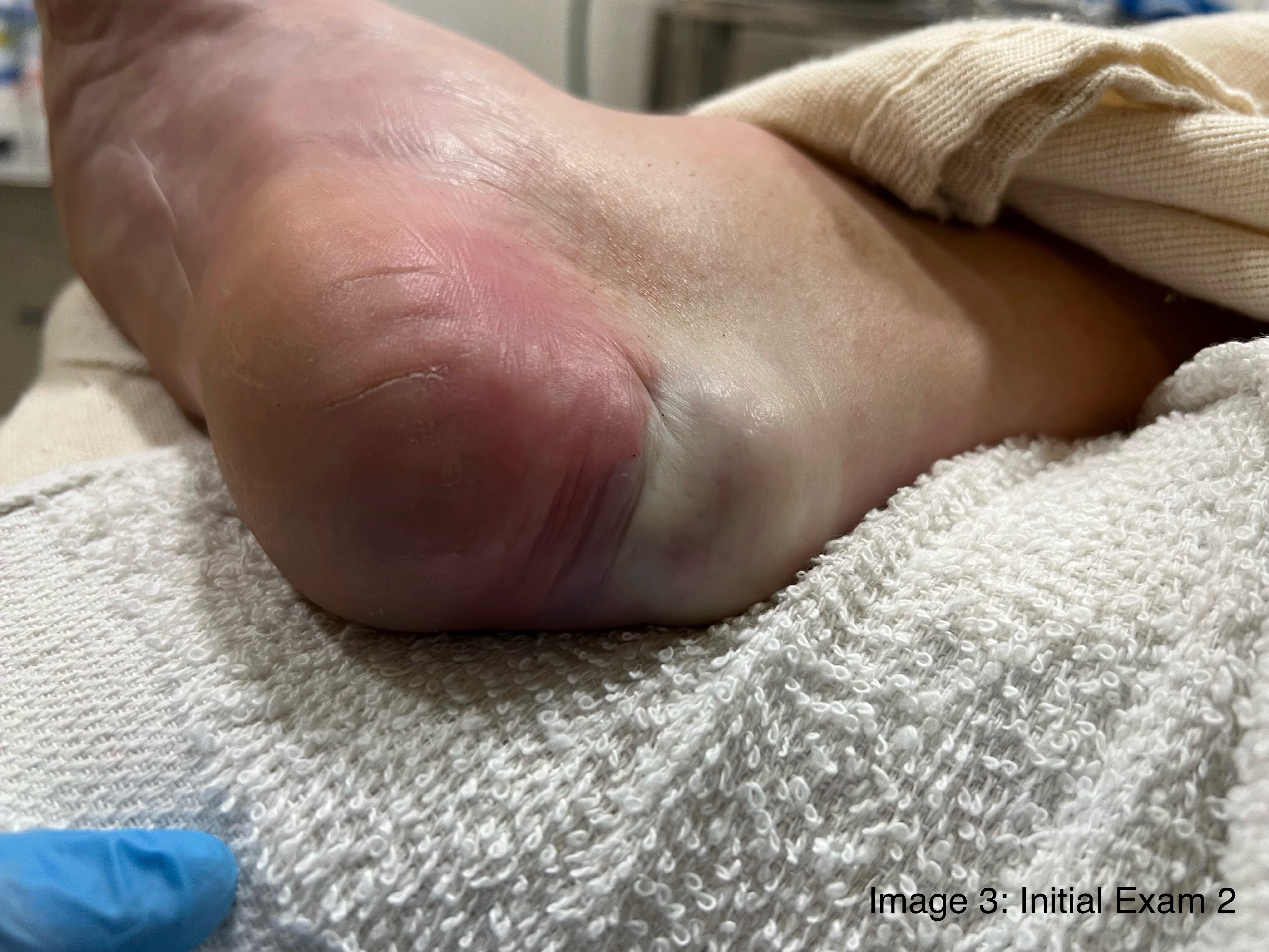

Supplement: Supplementary file 5 [file jetem-8-1-v28-supp5.jpg]

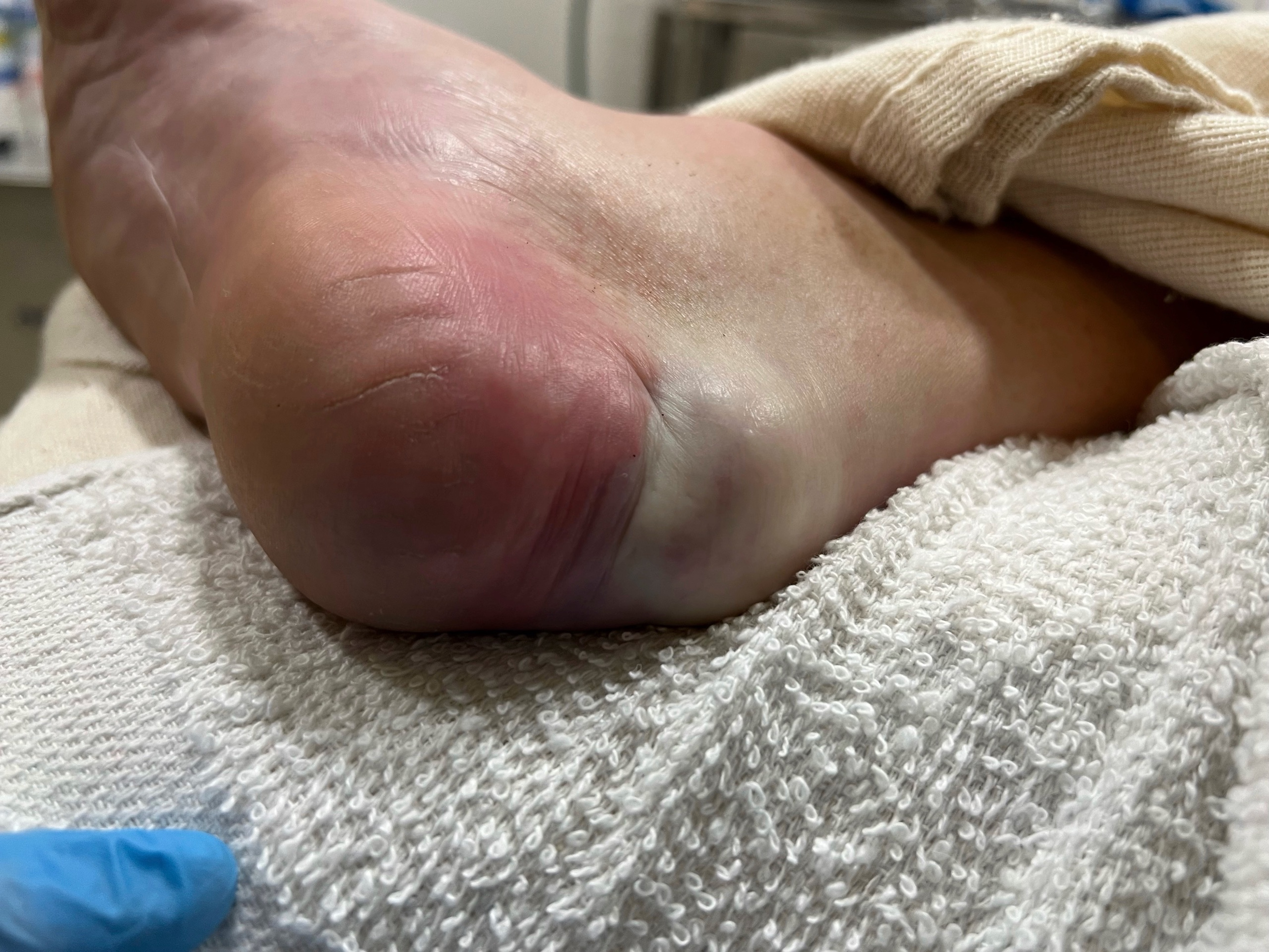

Supplement: Supplementary file 6 [file jetem-8-1-v28-supp6.jpg]

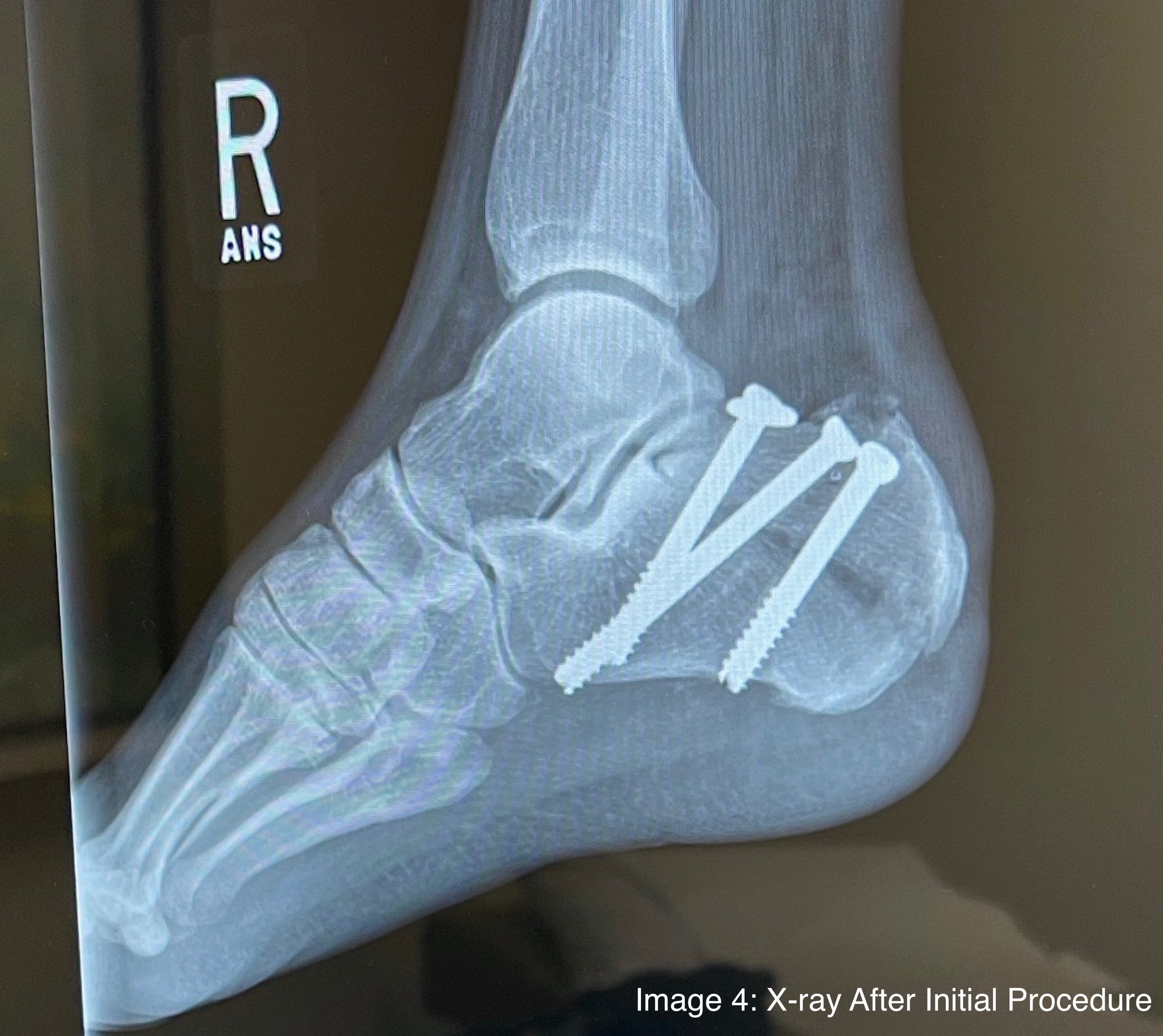

Supplement: Supplementary file 7 [file jetem-8-1-v28-supp7.jpg]

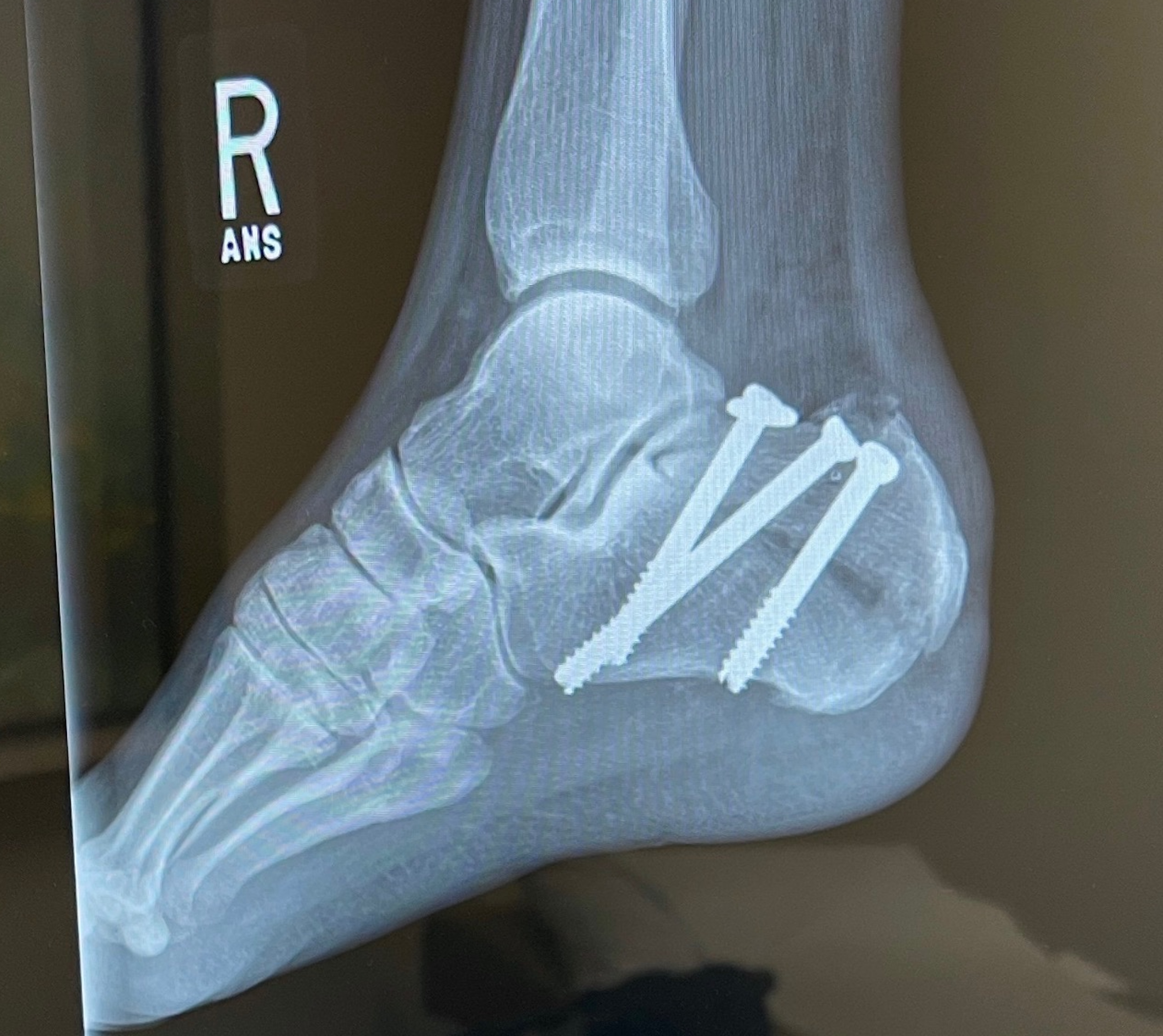

Supplement: Supplementary file 8 [file jetem-8-1-v28-supp8.jpg]

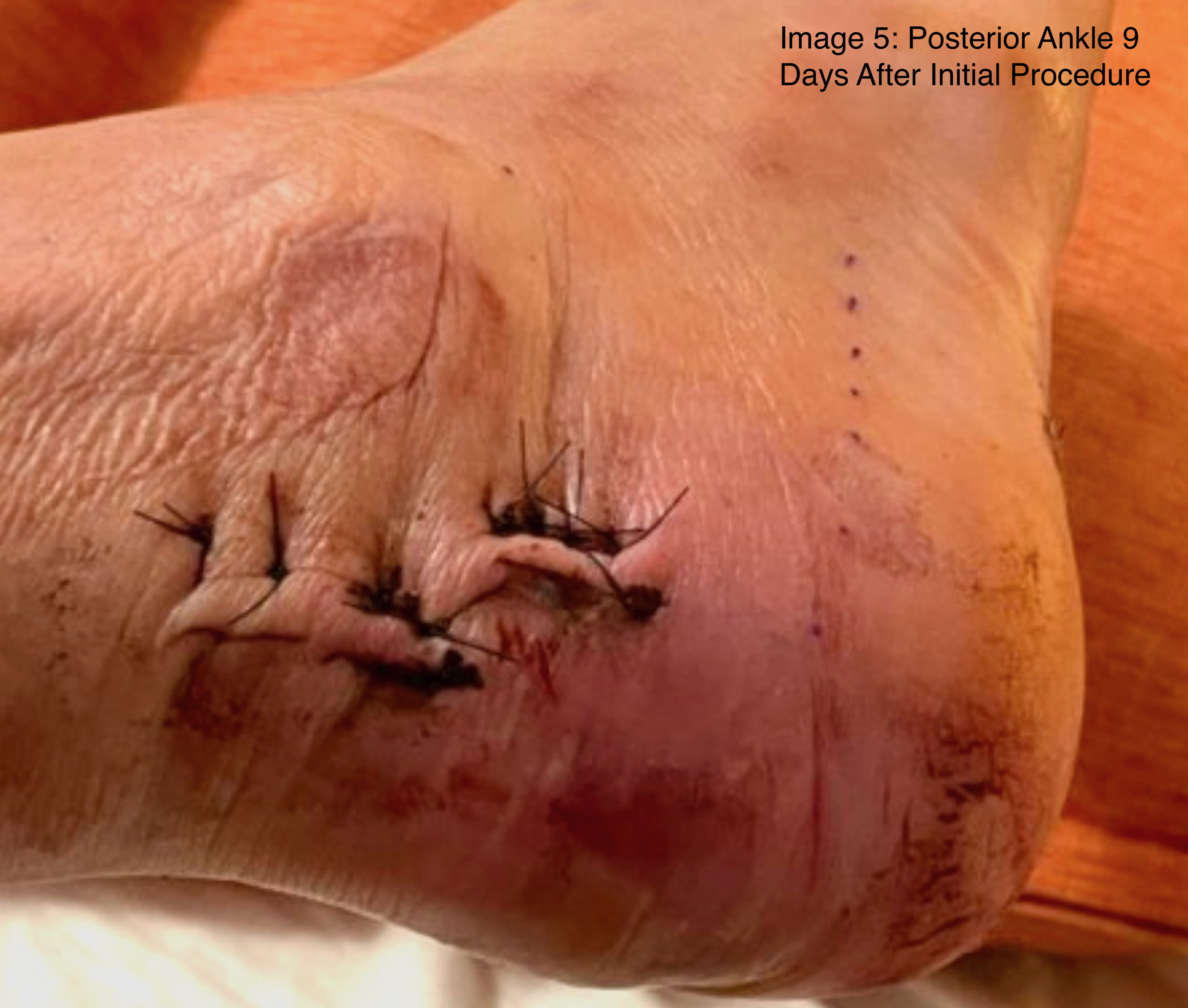

Supplement: Supplementary file 9 [file jetem-8-1-v28-supp9.jpg]

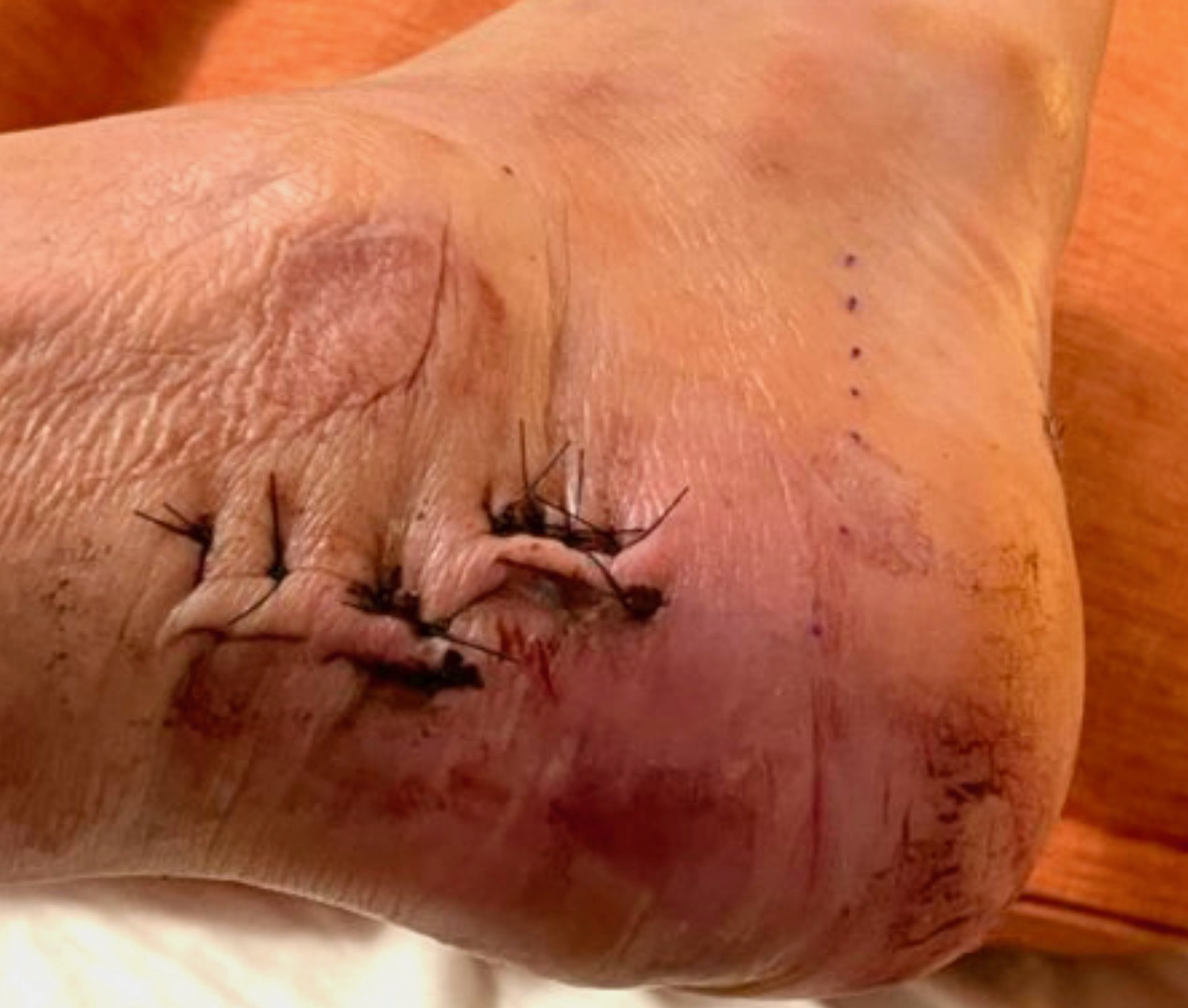

Supplement: Supplementary file 10 [file jetem-8-1-v28-supp10.jpg]

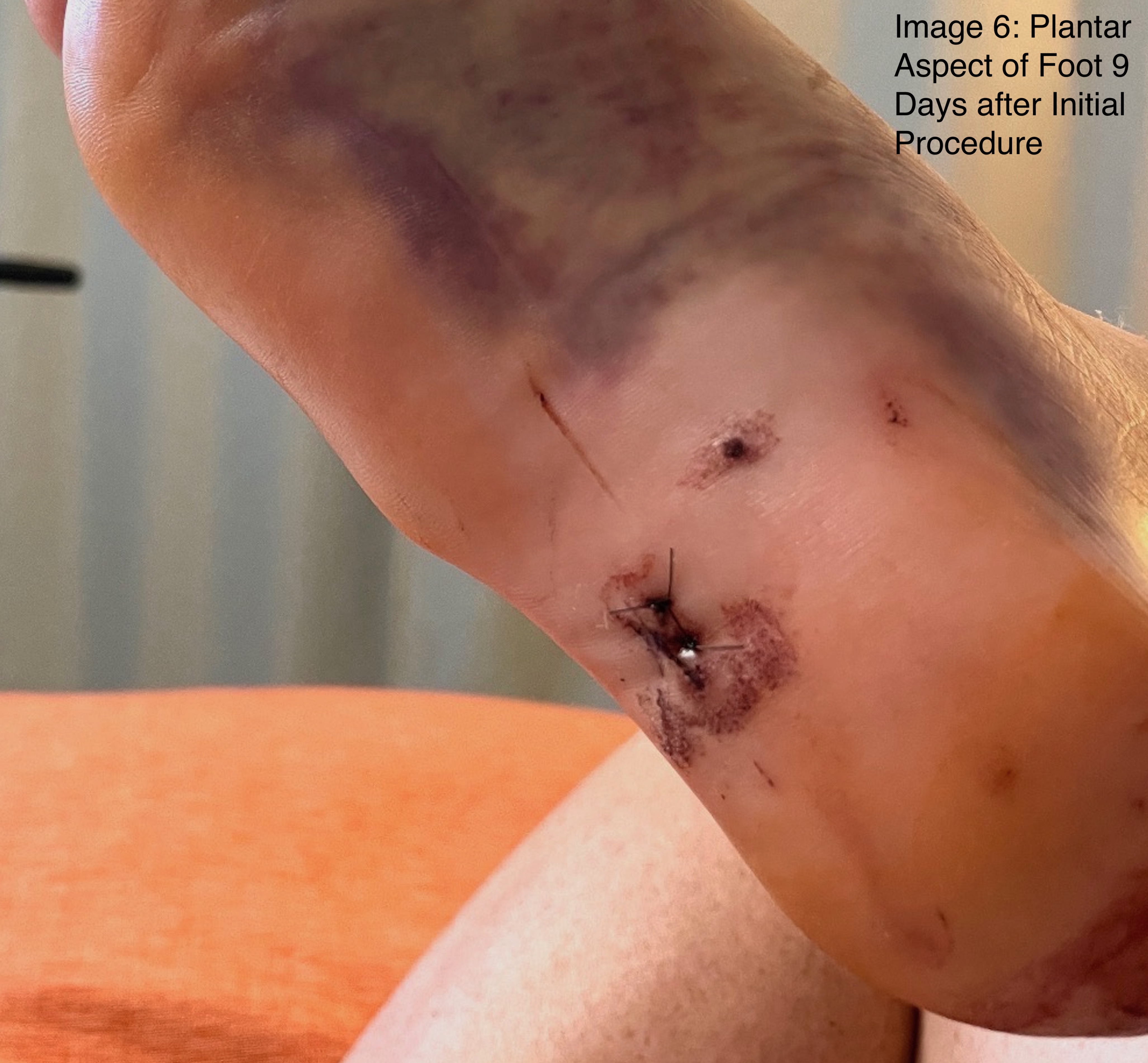

Supplement: Supplementary file 11 [file jetem-8-1-v28-supp11.jpg]

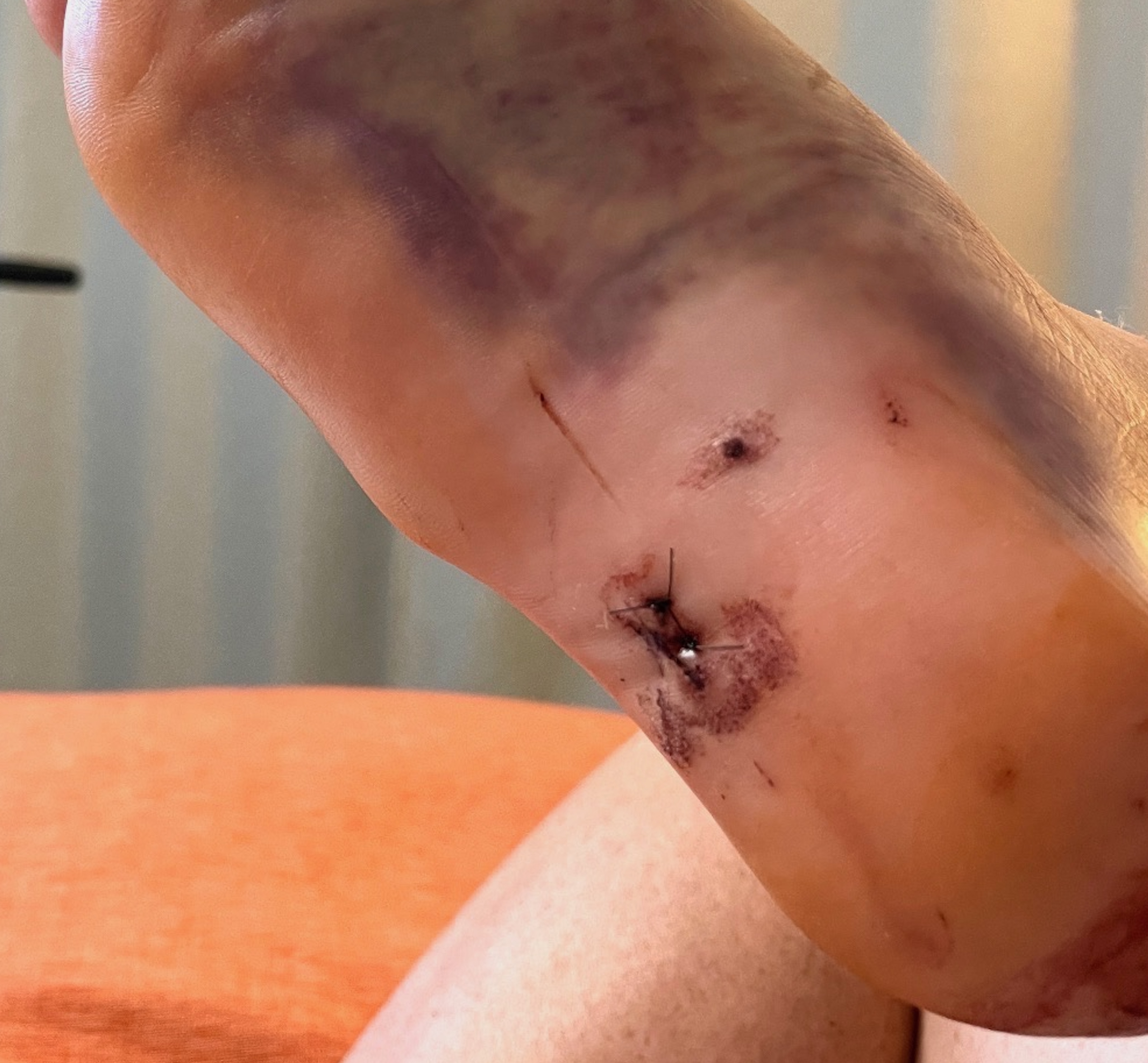

Supplement: Supplementary file 12 [file jetem-8-1-v28-supp12.jpg]

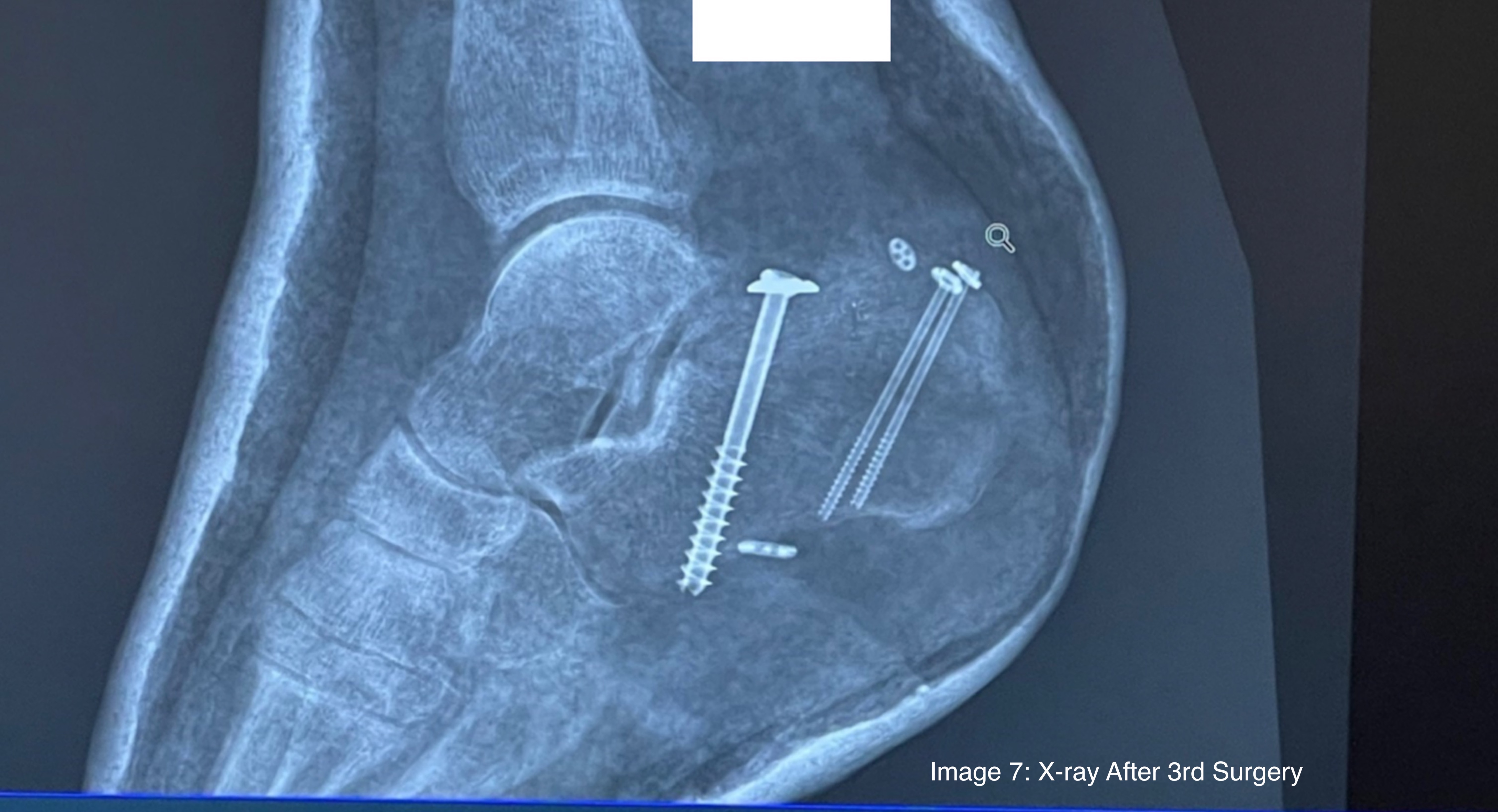

Supplement: Supplementary file 13 [file jetem-8-1-v28-supp13.jpg]

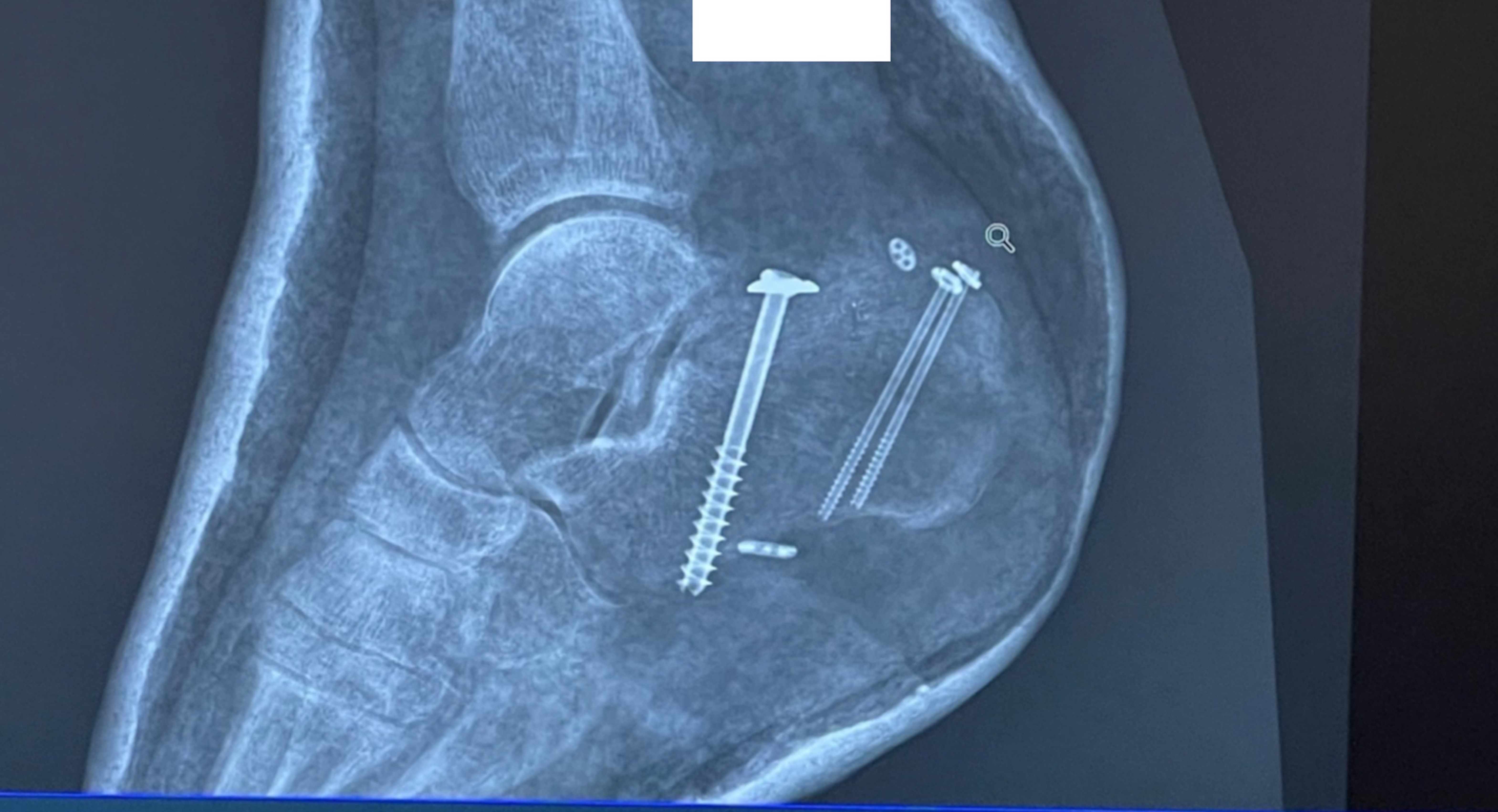

Supplement: Supplementary file 14 [file jetem-8-1-v28-supp14.jpg]

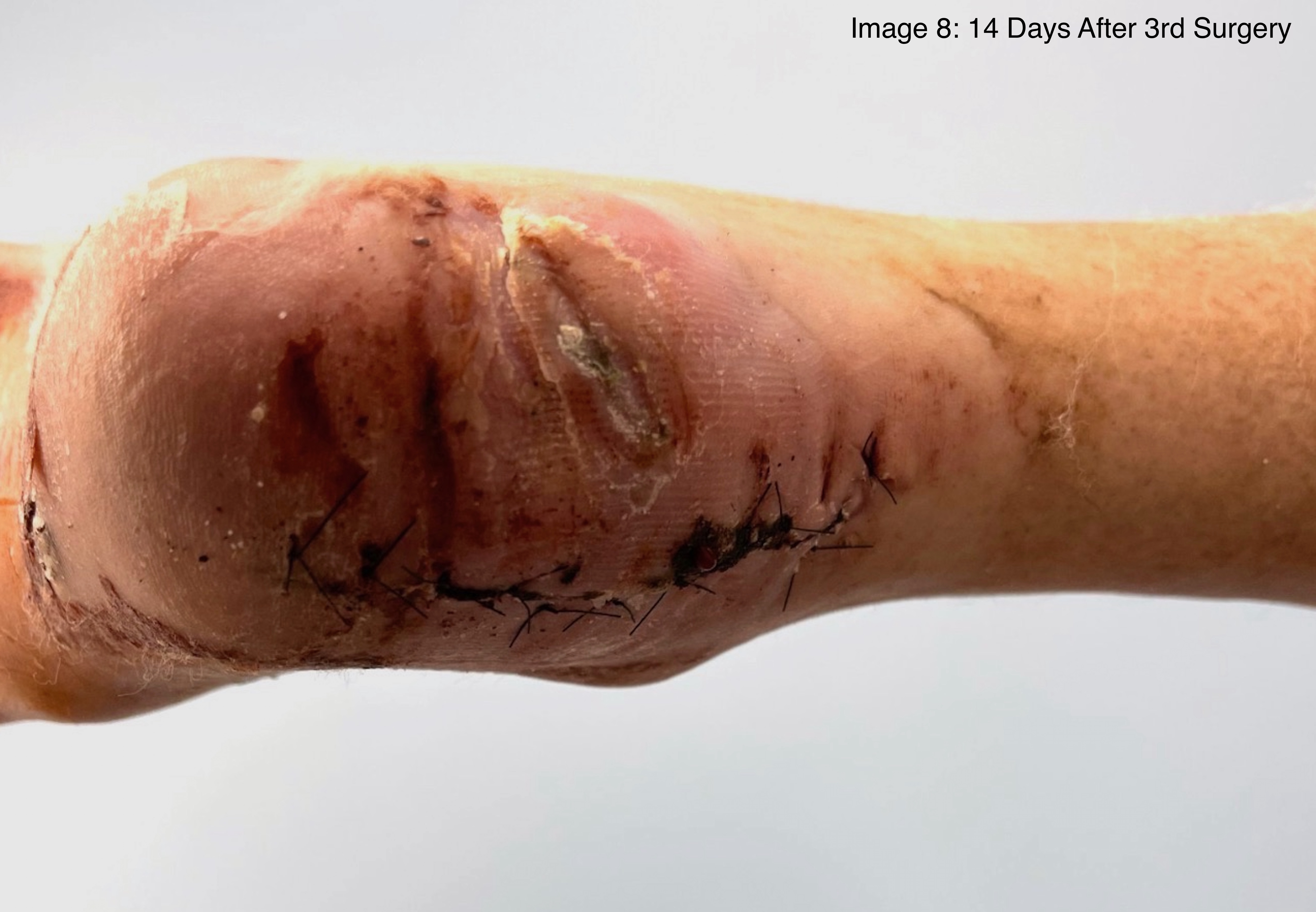

Supplement: Supplementary file 15 [file jetem-8-1-v28-supp15.jpg]

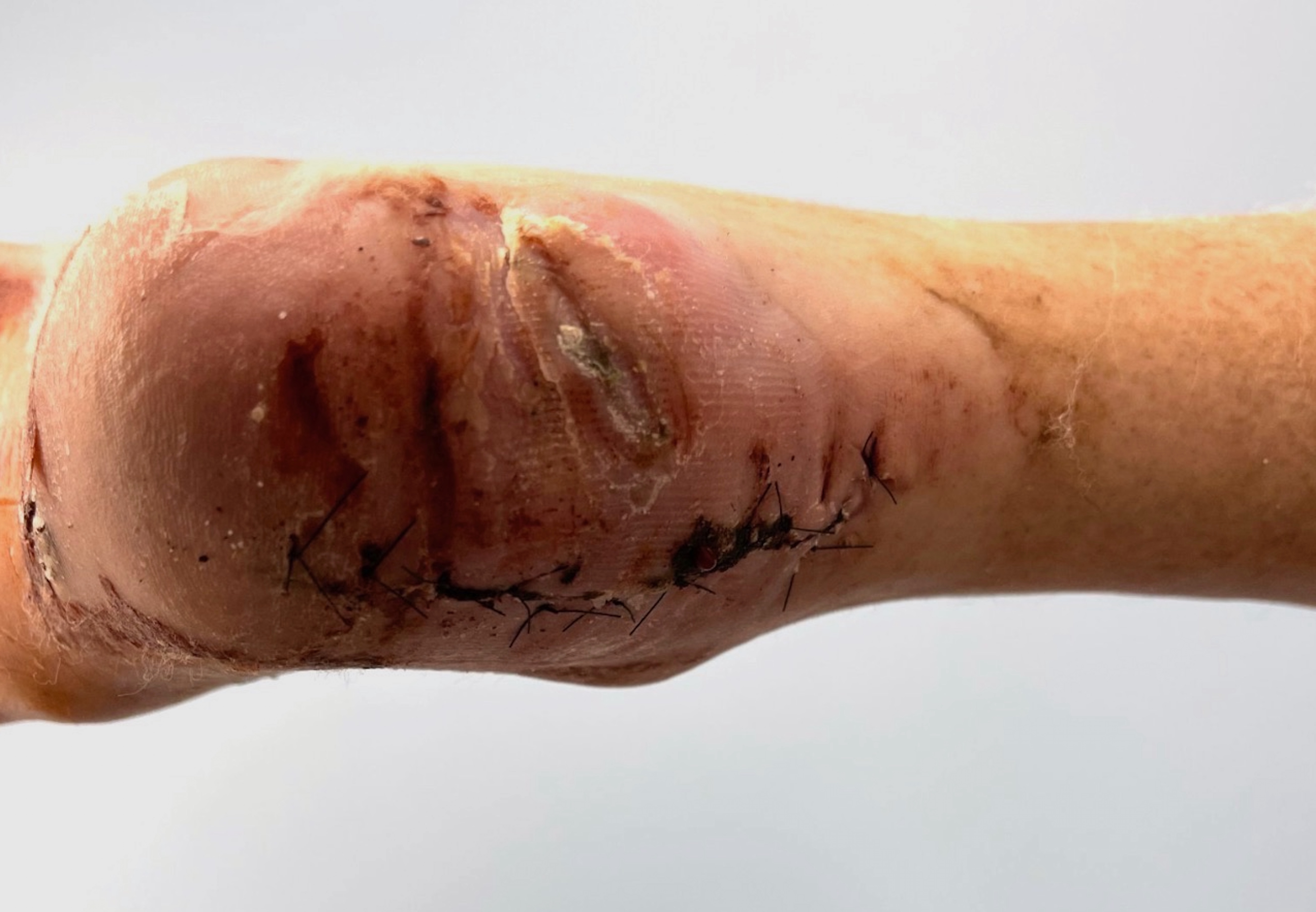

Supplement: Supplementary file 16 [file jetem-8-1-v28-supp16.jpg]

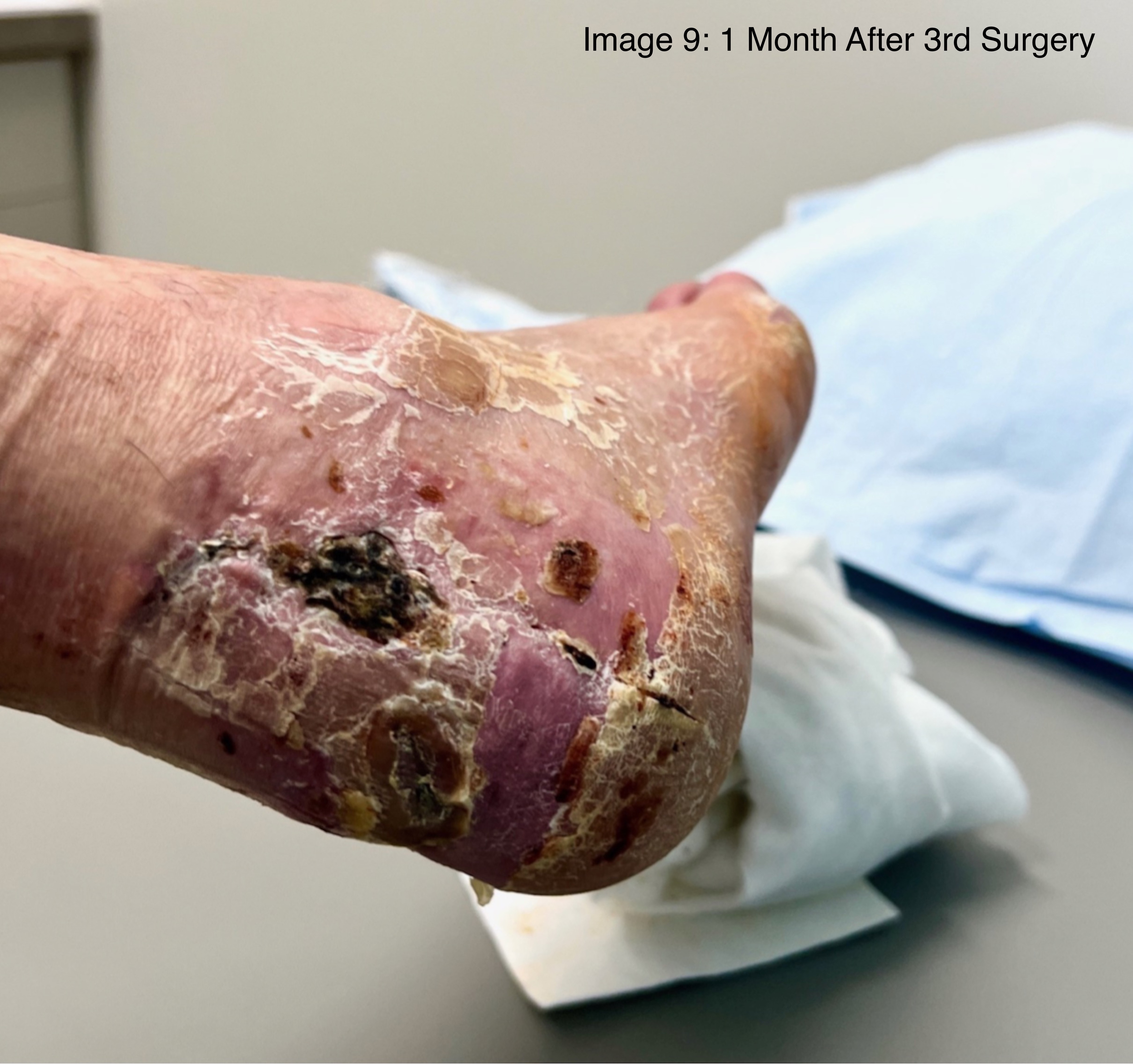

Supplement: Supplementary file 17 [file jetem-8-1-v28-supp17.jpg]

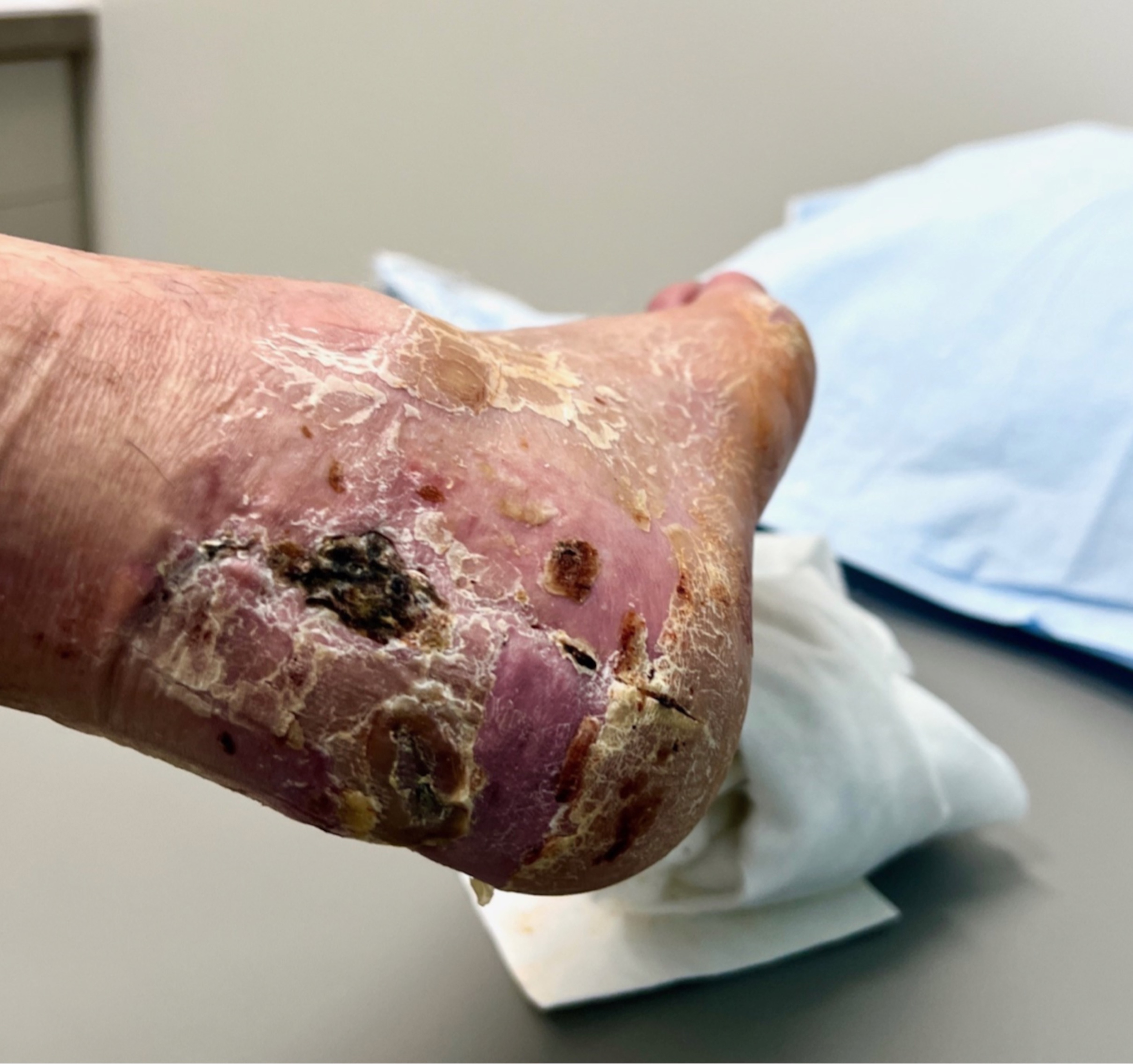

Supplement: Supplementary file 18 [file jetem-8-1-v28-supp18.jpg]

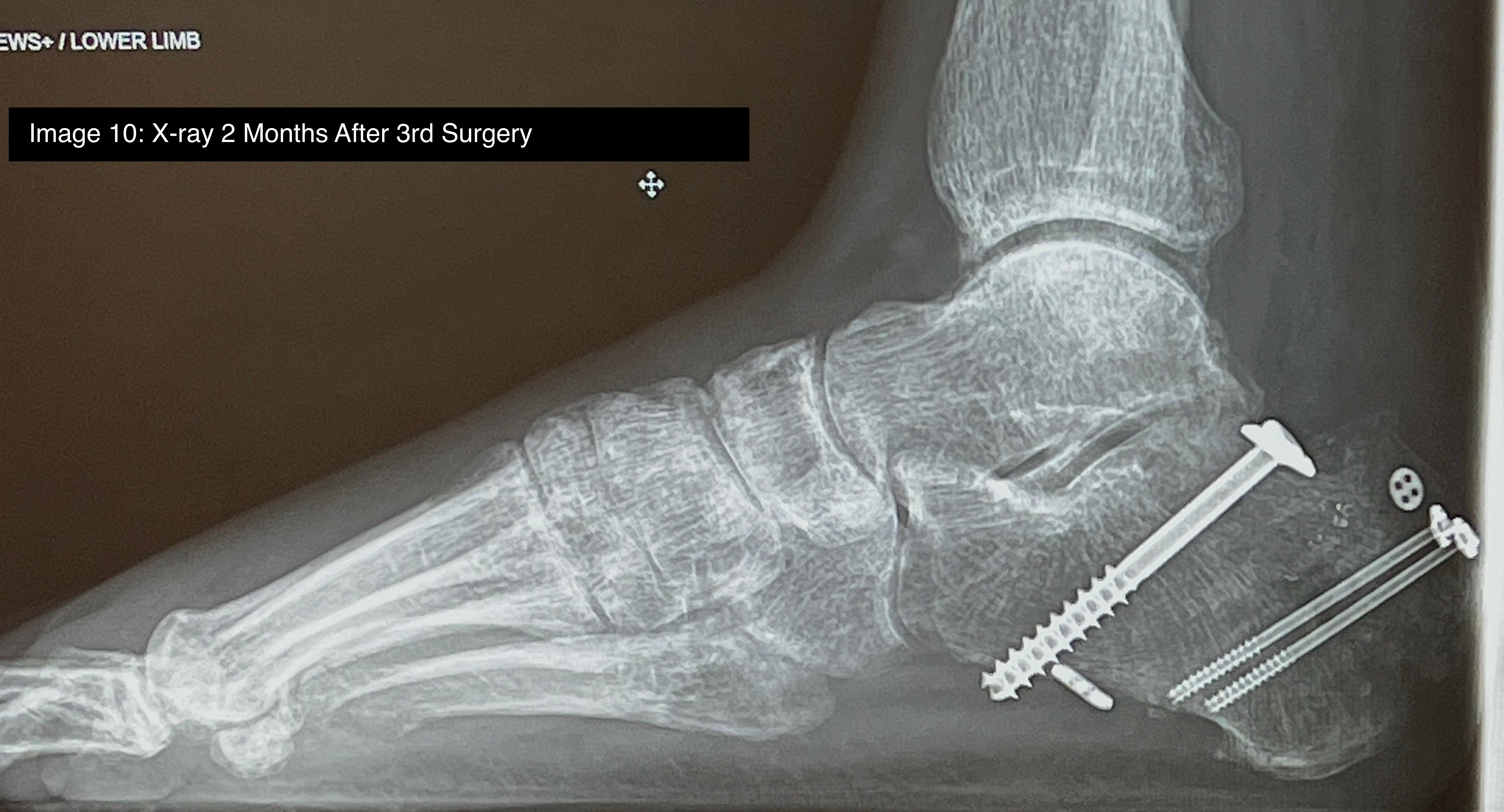

Supplement: Supplementary file 19 [file jetem-8-1-v28-supp19.jpg]

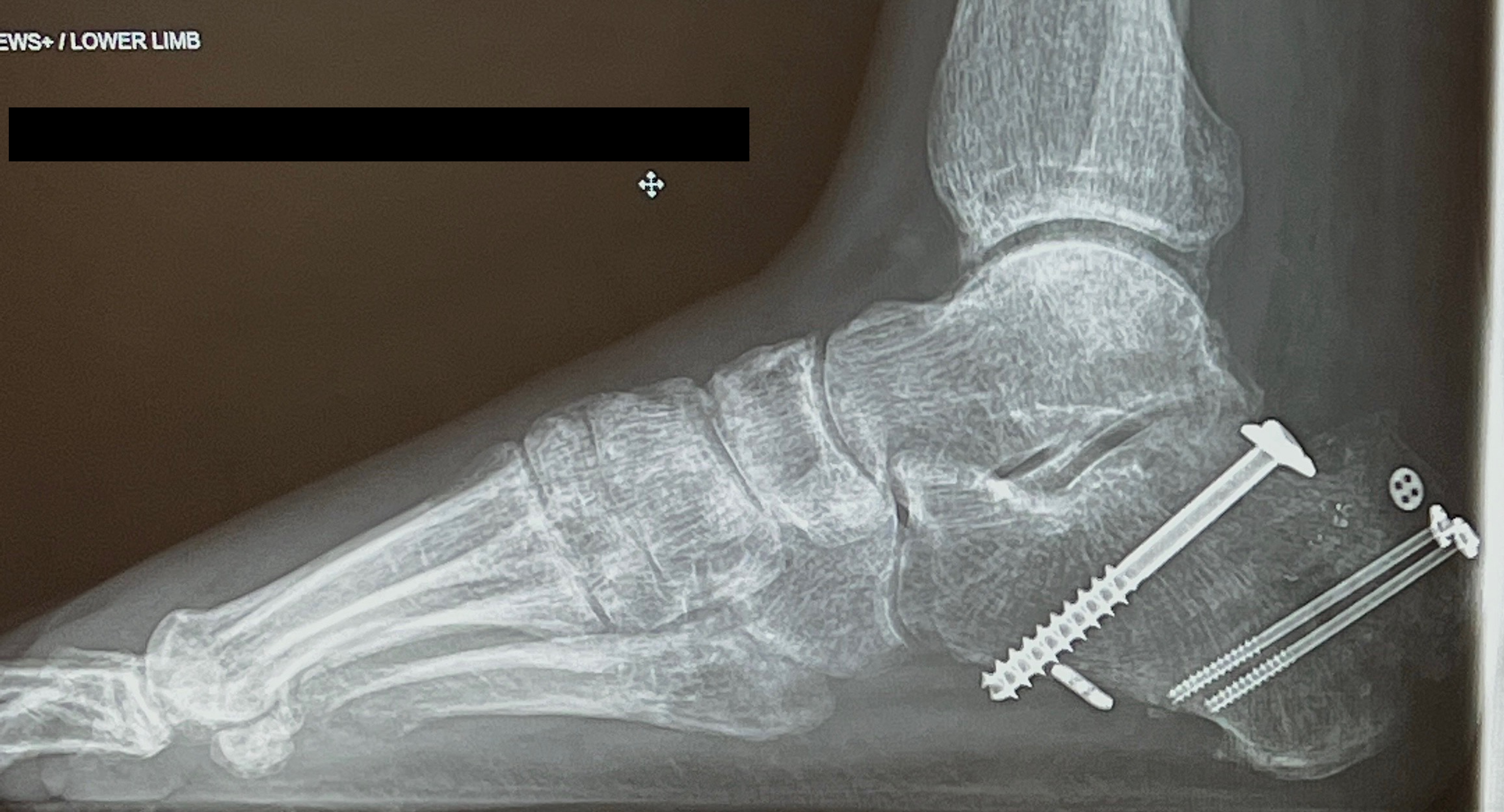

Supplement: Supplementary file 20 [file jetem-8-1-v28-supp20.jpg]

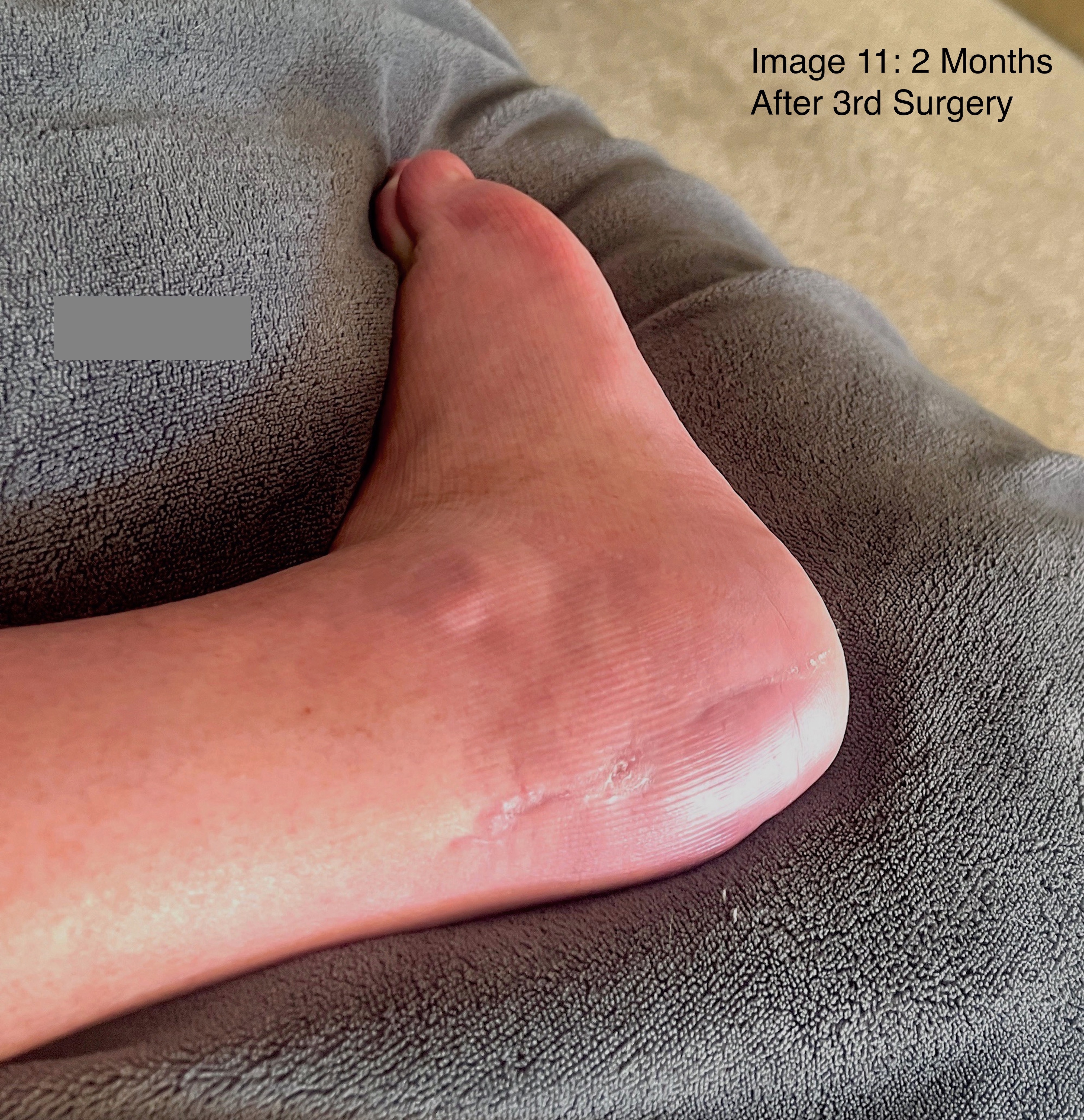

Supplement: Supplementary file 21 [file jetem-8-1-v28-supp21.jpg]

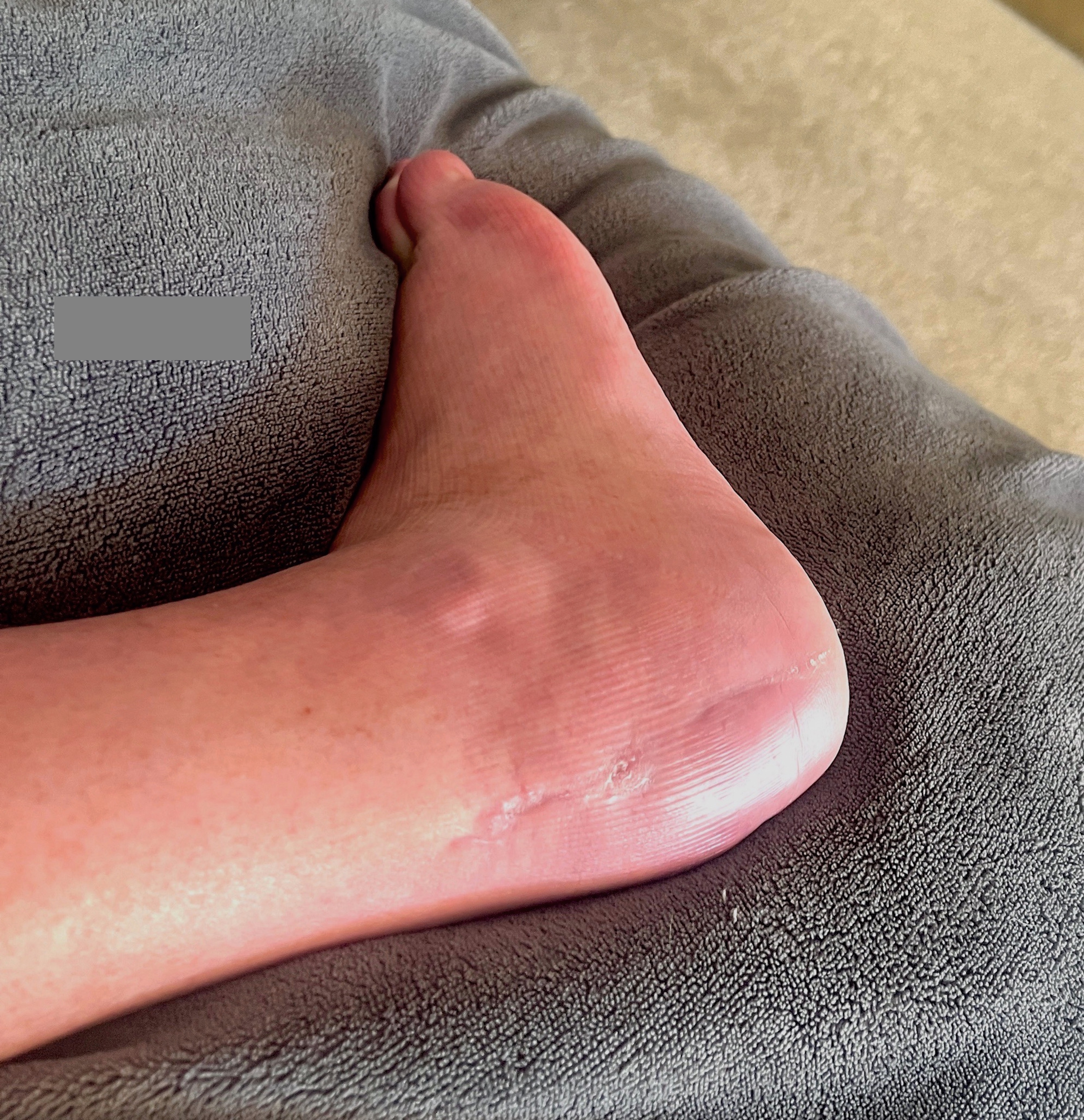

Supplement: Supplementary file 22 [file jetem-8-1-v28-supp22.jpg]
